# InvertNet: a new paradigm for digital access to invertebrate collections

**DOI:** 10.3897/zookeys.209.3571

**Published:** 2012-07-20

**Authors:** Chris Dietrich, John Hart, David Raila, Umberto Ravaioli, Nahil Sobh, Omar Sobh, Chris Taylor

**Affiliations:** 1 Illinois Natural History Survey, Prairie Research Institute; 2 Department of Computer Science; 3 Department of Electrical and Computer Engineering; 4 Beckman Institute for Advanced Science and Technology, University of Illinois, Champaign, IL 61820 USA

**Keywords:** Collection digitization, collection database, image processing

## Abstract

InvertNet, one of the three Thematic Collection Networks (TCNs) funded in the first round of the U.S. National Science Foundation’s Advancing Digitization of Biological Collections (ADBC) program, is tasked with providing digital access to ~60 million specimens housed in 22 arthropod (primarily insect) collections at institutions distributed throughout the upper midwestern USA. The traditional workflow for insect collection digitization involves manually keying information from specimen labels into a database and attaching a unique identifier label to each specimen. This remains the dominant paradigm, despite some recent attempts to automate various steps in the process using more advanced technologies. InvertNet aims to develop improved semi-automated, high-throughput workflows for digitizing and providing access to invertebrate collections that balance the need for speed and cost-effectiveness with long-term preservation of specimens and accuracy of data capture. The proposed workflows build on recent methods for digitizing and providing access to high-quality images of multiple specimens (e.g., entire drawers of pinned insects) simultaneously. Limitations of previous approaches are discussed and possible solutions are proposed that incorporate advanced imaging and 3-D reconstruction technologies. InvertNet couples efficient digitization workflows with a highly robust network infrastructure capable of managing massive amounts of image data and related metadata and delivering high-quality images, including interactive 3-D reconstructions in real time via the Internet.

## Background and introduction

Invertebrate collections present one of the greatest challenges to automated specimen digitization. Not only do they represent the majority of known species and comprise the largest numbers of available specimens, but they also present a number of logistical problems that have, so far, frustrated attempts to develop automated digitization and data capture workflows.

Insect collections, which constitute the largest extant collections of invertebrate specimens, are particularly challenging. In most, a majority of the prepared specimens are pinned. Dry, pinned insect specimens, when properly housed and protected from direct sunlight, high humidity and pests (e.g. dermestid beetles), may last indefinitely. Many European museum collections include pinned specimens collected centuries ago that remain intact and useful for comparative morphological study. However, even recently collected pinned insect specimens are often extremely fragile and easily damaged through handling. Moreover, to conserve space, many curators have packed specimens very densely into unit trays and drawers, such that adjacent specimens are nearly touching each other or even overlapping. Thus extreme care must be taken in moving specimens because legs, wings or antennae may easily be broken off if specimens are brushed against one another.

Specimen data obtainable from pinned insect specimens consist of the information (morphological and otherwise) embodied in the specimens themselves, and data (metadata) printed on one or more small data labels attached to the pin below the specimen. Specimen labels include information such as the collection locality, date, and name of collector, and the determined scientific name. These may be difficult to read and interpret because of their small size, the use of non-standard abbreviations, illegible hand-writing, and/or because they may be partly or completely obscured from above by other labels and/or by the specimen itself.

The traditional approach to digitization of insect collections (reviewed by Johnson 2007, 2009) has focused almost entirely on label data capture, retrospective georeferencing, and the assignment of unique identifiers to individual specimens. The usual workflow involves manually keying in data from specimen labels and attaching a unique identifier label (machine-readable barcode and/or human readable number) to each specimen. This approach is problematic for several reasons. It is time-consuming--one reason why so many existing specimens still need to be digitized. It is expensive, with per-specimen costs estimated at US$1 or more in some recently completed or ongoing projects (Vollmar et al. 2010, Heidorn 2011 and unpublished data). It is error-prone, with typographical or other mistakes often introduced during the process of label data interpretation and transcription. It also entails substantial risk of specimen breakage due to handling, particularly if the work is being performed (as it often is) by poorly paid student technicians with little collection management experience.

Thus, the major challenges for InvertNet and similar projects are to bring the per-specimen cost of digitization down without sacrificing accuracy of data capture or risking damage to irreplaceable specimens. Indeed, the NSF ADBC program, the source of funding for InvertNet, mandates that the average cost per specimen for digitization, program-wide, including both imaging and label data capture, be kept at or below US$0.10. ADBC aims to digitize 1 billion specimens in 10 years for a total budget of US$100 million.

Despite the problems noted above, one aspect of pinned insect collections that may prove advantageous to automated mass-digitization methods is that the specimens are usually mounted and arranged in a consistent orientation and multiple specimens of the same taxon are usually grouped together, side-by-side, within the same collection storage unit. Thus, high-resolution digital imaging methods can be used to capture images of large numbers of specimens simultaneously, thereby drastically reducing the per-specimen cost of obtaining specimen images. Other recent projects have already used this approach to acquire images of collections of pinned specimens very quickly and cheaply (Bertone and Deans 2010 and this volume, Blagoderov et al. 2010 and this volume). Immediate access to the images may then be provided via the Internet, which, in turn, may facilitate at least partial acquisition of specimen metadata (i.e., label data) by the broader community of potential users.

Some problems remain to be addressed, however. These include the need to acquire specimen-level label data and to assign unique identifiers that allow individual specimens to be tracked. Top-down images of whole drawers of pinned insects allow users to view some specimen label data, but labels are often at least partly obscured by the specimens. In cases where series of specimens from the same collection lot are placed together, it may be possible to assemble all the label data by examining different specimens in the series because different parts of the labels of different specimens may be visible. 3-D reconstructions that allow virtual tilting of drawers or specimens may reveal parts of labels obscured in a strictly top-down view. Unfortunately, even 3-D reconstructions will not allow labels placed beneath the top label on the pin to be viewed if the labels are pushed together. Use of even more advanced technologies such as micro CT scanning may eventually allow data to be captured from labels that are completely obscured by specimens or other labels, but at present, such data are accessible only through physical manipulation of specimens and labels. In such cases, the added value of gleaning this additional information needs to be balanced against the risk to the specimen posed by physical handling. Fortunately, for most specimens, a large proportion of the crucial occurrence data are printed on the top label and, because most insect specimens are small, these labels may be read without physically manipulating the specimens themselves. Examination of gigapan images (see gigapan.org) of whole drawers of pinned insects from the North Carolina State University insect collection indicates that more than 75% of the drawers and ca. 90% of the specimens imaged have text on the top label visible; this label usually comprises at least the locality name and, in most cases, also the date of collection and name of collector. Because the arrangement of pinned specimens in the NCSU collection is typical for insect collections in general (at least in the USA), large amounts of species occurrence data should be obtainable directly from high quality images of entire drawers. We estimate that 3D reconstructions that allow virtual tilting of images with similar resolution will increase the amount of label data exposed by at least 50%, i.e., by exposing more of the top label when it is partly concealed by the specimen and by exposing labels attached farther down on the pin.

Specimen tracking is another problem that may be difficult to overcome with mass specimen digitization approaches. Recently it has become standard practice for curators to attach separate barcode or other unique identifier (UID) labels to individual specimens as part of the specimen data capture/digitization workflow (Johnson 2009). In our view, the risk of specimen damage posed by attaching such labels may outweigh the need to uniquely identify each individual specimen, especially if the specimen is being handled *only* for the purpose of attaching the barcode label. A better approach might be to attach UID labels to specimens only when the specimens need to be handled for another purpose, e.g., when being transferred into a shipping container during loan processing, or when being sorted and identified by a taxonomist or curator. Because the only value of attaching a physical UID label to the individual specimen is to facilitate tracking of the specimen after it has been moved from its original location in the collection, we recommend that curators not add UID labels to specimens until they need to be moved for other reasons. Prior to being moved, individual specimens in digitized drawers and unit trays may be digitally mapped based on their physical locations. A specimen record may then be created in the collection database and include a unique identifier and information on its location, in addition to data from the specimen labels. The unique identifier, thus assigned, will remain a virtual UID until the specimen needs to be moved, at which point a physical label may be printed and attached to the specimen. Alternatives to ink-on-paper UID labels, such as passive Radio Frequency Identifier (RFID) tags (which may be pinhead sized and have recently become quite affordable) should also be explored. Because RFID tags (unlike barcodes) do not need to be visible in order to be detected and scanned, they offer the added advantage of further reducing the need for physical manipulation of specimens. They also offer the possibility of developing Augmented Reality (AR) systems capable of physically mapping the locations of specimens in three-dimensional space (e.g., within a drawer, cabinet or collection range) using radio telemetry.

### Recent advances in high-throughput insect specimen imaging

Most recent collection digitization initiatives that include an imaging component have focused on capturing images of individual specimens (e.g., Lampe et al. 2005, Enriquez 2011, Ball et al. 2011, Eades et al. 2012, Harman et al. 2011, Haüser et al. 2005, Kjar et al. 2012). While this approach may have the potential advantage of producing very high-quality images of individual specimens, it also requires physical manipulation of the specimens, which entails risk of specimen damage and has a high per-specimen cost. Most digitization initiatives that have adopted this approach have focused only on high value collection holdings (e.g., type specimens). A cost/benefit analysis of this approach needs to be undertaken, since the risk of damaging such specimens during the digitization process must be weighed against the benefits gained by providing access to the digital images (e.g., how often is a particular research need addressed by access to the image alone, rather than to the specimen itself?). If such high quality images of individual specimens are being captured for other purposes (e.g., for publication in a taxonomic paper), they should be archived and associated with the collection database record for that particular specimen.

Recent advances in digital gigapixel imaging allow images of entire drawers of pinned insects to be captured. Multiple neighboring images can be “stitched” together into a single “panoramic” image. This stitching operation is enabled by recent advances in computer vision, and relies on finding matching features in the overlapping regions shared by neighboring images. By capturing multiple high resolution images and stitching them together into a single panorama, drawers containing thousands of specimens may be digitized very rapidly and the quality of the final images may be very high. This method was used successfully at North Carolina State University (NCSU) in a recent NSF-funded project (Bertone and Deans 2010) and suggests a promising pathway toward more efficient methods for mass imaging and digitization of pinned insect (and other) collections.

Using the GigaPan robot (gigapan.org) combined with a consumer-grade digital camera, the NCSU team was able to capture images of their entire collection, comprising >2700 drawers within just a few person/months and make these high quality images available to the public via the GigaPan website. The web interface allows users to view images of entire drawers and zoom in onto individual specimens, such that label data (when not obscured from above by large specimens) and details of the morphology of the specimens may be seen. Hand-entering data for each of the approximately 2 million specimens in the NCSU collection, using traditional methods, would have required many person-years of effort. The GigaPan project provided rapid access to the entire collection.

One problem with the NCSU/GigaPan digitization methodology is that it provides only limited access to specimen label data (capture of label data was not one of the stated goals of the project). Only the label data not obscured by the specimens may be extracted from the GigaPan images and the data are neither available as text, nor have they been parsed into the standard Darwin Core database fields (http://rs.tdwg.org/dwc/) to facilitate automated searching of particular data elements. Another problem is the distortion introduced into the stitched gigapixel images caused by the fixed position of the robot-mounted camera over the center of the drawer. During image capture, the robot tilts the camera from front to back and side to side, such that the edges of the drawer are photographed at an angle while the center of the drawer is photographed with the lens pointing directly downward. The resulting stitched images show a pronounced fish-eye effect (barrel distortion) with the sides of the drawer bowed outward. Stitching software (e.g., Hugin open-source stitcher; http://hugin.sourceforge.net/) exists that includes tools to correct for this distortion to some extent, but it is difficult to remove all distortion from the stitched image if the original images from which the stitched image is constructed are themselves highly distorted.

The SatScan system implemented at the Natural History Museum, London (Blagoderov et al. 2010), uses an alternative technology that overcomes the distortion problem. In this system, the camera does not tilt but moves horizontally, capturing images all from the same angle but at different X/Y positions over the drawer. Images produced by this system have similar levels of resolution to those obtained in the NCSU/GigaPan project, but the drawer images produced by SatScan are free of distortion, even toward the edges of the drawer (http://sciaroidea.info/node/44309).

Still the problem of capturing label data persists. Although labels attached to insect specimens are usually very small, most insects are also small, so, for a large proportion of pinned specimens in collections, label data are at least partially visible from above. As any insect taxonomist knows, it is usually possible to see more (sometimes all) of the label(s) simply by tilting the drawer or otherwise viewing the specimens from an angle. This can be seen in many of the NCSU GigaPans (http://www.gigapan.org/profiles/ncsuinsectmuseum), where the labels of specimens toward the edges of the drawers are more exposed than those near the center, simply as an artifact of the GigaPan image capture protocol. An improved system that maximizes visibility of the labels, in situ, would simply need to capture images of the drawer from multiple perspectives, including different horizontal positions over the drawer (*à la* SatScan) as well as different angles (*à la* GigaPan). Technologies for combining such images to create 3-D reconstructions can then be used to allow virtual tilting, maximizing the user’s ability to read the data on labels partly obscured by the specimens or by other labels. This is the approach we envision using for InvertNet.

## The InvertNet approach

Our efforts to implement robust, rapid and cost-effective solutions for mass digitization of invertebrate collections focus on four main areas: 1) use of improved image capture hardware; 2) application of improved image processing and visualization methods; 3) development of user-friendly, semi-automated workflows; and 4) establishment of robust cyberinfrastructure for data ingest, storage and delivery.

### Improved image capture hardware

The primary goals of an ideal capture system include:

1. The system should be as automated as possible to minimize operator activity and therefore human error;

2. It should capture an array of high resolution images from multiple viewpoints, to support zooming in to reveal specimen detail, viewing otherwise occluded portions of pin labels, and 3-D reconstruction;

3. It should be inexpensive to purchase, operate, and maintain;

4. It should be flexible to adapt to operator and scientific feedback from operations when deployed.

5. It should be upgradable once deployed, to take advantage of improvements in imaging technologies (sensors, processing, etc.) as they become available.

We have investigated three options for capturing such images. We first investigated combining multiple GigaPan-style panoramas from different viewpoints, such as from four corners of the specimen drawer, and using prost-processing to create composite images. However, this can increase time, effort, and the probability of human error if the drawer and/or camera must be re-positioned manually during processing of a single tray.

A more reliable option used a robotic camera positioning system based on a modified Computer Numerical Control (CNC) machine (similar to a plotter, except with the pen replaced by a camera) to position camera/lens precisely and repeatably in an x-y grid to complete the panorama. Such robotic systems are capable of moving tools (including cameras) rapidly and precisely in three dimensions offer great advantages in terms of adaptability by programming various capture “recipes” based on tray geometry, specimen layout, and specimen scale and density within a tray.

In order to minimize distortion between neighboring images and reduce stitching artifacts, we use a telecentric lens that captures an orthographic (not perspective) projection of the image on the sensor. The telecentric lens shoots the same image area regardless of how far away it is, and one cannot enlarge or reduce the area being photographed by moving the camera closer or farther away. This is beneficial for measurements, image processing and stitching, but precludes the use of neighboring image overlap processing for multiple view (3-D) and occluded label processing.

To accommodate these multiple viewpoints, we extended the CNC camera positioning machine with a computer controlled pan-tilt mechanism that provides the ability to capture grids of overlapping images at various positions, and also at various oblique angles in order to simultaneously support accurate panorama generation, 3-D reconstruction, and occluded label capture.

CNC systems were developed for machining dense materials with industrial power tool heads. They are large and heavy, often hundreds of pounds. Furthermore the physical size of the moving parts of such machines complicates lighting, as large machine parts move through the path of lighting sources during capture, altering lighting conditions and casting shadows which can affect feature matching algorithms such as panoramic image stitching. Because of their industrial development for machining, CNC machines are large and not able to be easily disassembled, massive – hundreds of pounds, require high power, and are not easy to move, ship, and locate in a laboratory setting.

We are currently testing a more lightweight prototype that is based on the Delta Robot (http://en.wikipedia.org/wiki/Delta_robot), which resembles a three-legged spider that suspends the camera over the specimen drawer, with much less hardware to interfere with fixed lighting systems. These robotic systems are very fast and accurate, and are used in “pick and place” factory lines for purposes such as picking items and aligning them for packaging. Such a machine is inexpensive to build and can be programmed to accomplish very rapid, precise and complex movements (for example see: https://www.youtube.com/watch?v=foTE0Mau5a8). We are currently working with a 3-arm design with additional pan/tilt motors that allow the camera to be rotated in addition to precisely placed in x-y-z position over the drawer of pinned specimens. The machine is far less massive when compared to a CNC style system - tens of pounds, and is easily disassembled and reassembled without machinists tools and expertise. This facilitates shipping, lab positioning, movement, and physical requirements of the system.

### Stitching software

Software capable of combining multiple images into a single panorama is now widely available. The GigaPan software system, used successfully in the NCSU digitization project, is one example. One current disadvantage of the GigaPan software is that it requires that final, stitched images be posted to the GigaPan.org website in order to be viewed and manipulated via the Internet. Open-source stitchers (e.g., Hugin, OpenCV) and Zoomable User Interfaces (ZUIs) such as Zoomify, required to view and manipulate the image are now also available and provide greater flexibility for the development of customized interfaces and workflows (see below).

Stiching algorithms rely on feature detection and matching across the raw images, which can be computationally demanding for large numbers of images. Two of our team, Hart and Raila, are participants in the Illinois-Intel Parallelism Center (I2PC) which is focused on new multicore parallel computing architectures, techniques, and tools. In collaboration with I2PC we are exploring parallel implementations of stitching codes. Results to-date have shown order of magnitude performance increase (from 500 seconds to 40 seconds) on modern commodity desktop computer systems, and we believe that the next generation of processors should accelerate the performance to levels that should not impede the workflow of digitization when run on commodity systems, but the stitching codes are also able to be run on large scale super-computing systems within the server-side of the InvertNet infrastructure if needed.

### 3-D Reconstructions

In addition to providing a means for creating distortion-free 2-D gigapixel images of entire specimen drawers, by using advanced hardware to vary the viewpoint and direction of image capture, we enable two new and exciting capabilities. From different vantage points, we can better see beneath the specimens to better capture the data from the labels pinned below them, and images from multiple view directions can be used to reconstruct 3-D models of the specimens themselves, potentially facilitating capture of more morphological data than is possible using 2-D, top-down images. We have tested multi-view stereo (MVS) reconstructions on specimen capture images and reconstructed 3-D models from them. MVS takes a pair of photographs from two different viewpoints and “rectifies” them, distorting them so corresponding points in each image have the same “y” coordinate. It can then search along horizontal lines for these matching points and uses the disparity in their alignment to estimate their distance from the viewer. Such estimates can be error prone and require further smoothing. Our current MVS reconstruction is based on a state-of-the-art algorithm developed by Disney Research Zurich for reconstruction of human faces for feature film production (Beeler et al. 2010). However, the smoothing designed for facial geometry does not work well on the insect specimens tested so far and we are researching new methods that work better on the dark, sparse and fine features from high-resolution invertebrate images.

### Digitization of other kinds of specimen storage units

Invertebrate collections consist not only of pinned specimens stored dry in drawers and unit trays, but also include fluid (usually ethanol) preserved specimens in vials or jars, and specimens mounted on microscope slides. The methods described for capturing images of whole drawers may be extended to these other storage types. Images of multiple slides or jars may be captured simultaneously and then segmented to facilitate data capture for individual units. This is the approach taken by another project at the Illinois Natural History Survey and University of Minnesota, recently funded by NSF (Tinerella 2010). Slide mounted specimens are perhaps the easiest to digitize: they may be treated as two dimensional objects and, because they are of standard size, individual slides may be imaged in groups placed in fixed positions on a tray and then segmented using a simple pixel map of the tray. Once digitized, the specimens and labels are clearly visible on the image and the image may then be used as a surrogate for the physical slide during subsequent label data capture. Following this approach, InvertNet is capturing images of 20 slides at a time by arranging them in fixed positions on a clear plastic template placed on the bed of a consumer-grade flatbed scanner. Images captured in this way are of sufficient quality to reveal label text and the general condition of the specimens but, in most cases, not good enough to reveal details of specimen morphology sufficient for species identification or morphological study. A variety of automated systems are available commercially for digitizing collections of microscope slide-mounted specimens, combining robotic slide loaders with high quality microscopes or scanners (Rojo et al. 2006) but, to our knowledge, none have yet been applied to large-scale digitization of slides in natural history collections.

**Figure 1.**
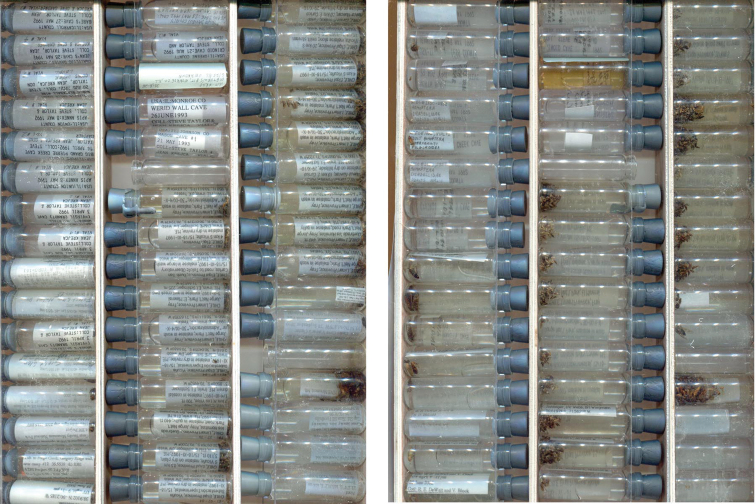
A set of three-dram vials scanned using a color flatbed scanner showing the front (left) and back (right) of the same set of vials. Note that the position of empty spacer vials (e.g., sixth from top in middle column) is the same, but inverted, in the two images because the vial racks are flipped vertically between scans. This relatively quick and inexpensive procedure exposes at least some label data for subsequent capture and reveals the general condition of specimens.

Fluid-preserved specimens in vials present a greater challenge. Multiple specimens are often stored in the same vial and the orientations of specimens and labels vary among vials. Views of vial contents are distorted by the refractive properties of the glass and fluid and the labels may obscure the specimens, or vice versa, to greater or lesser extent. Complete digitization of ethanol-preserved specimens now requires laborious removal of the specimens from the vials so that they may be spread apart and imaged. We are experimenting with methods for capturing images of multiple vials simultaneously. At present, the relatively low-cost proposed approach for InvertNet uses a flatbed scanner to capture images of multiple vials simultaneously using customized vial racks with clear sides. Racks containing vials are oriented so that as much as possible of the label(s) in each are in view, the racks are then placed on their sides on the scanner bed and scanned (Fig. 1). The racks are then flipped over (180 degrees vertically) to capture a second image of the opposite sides of the vials. This approach allows entire collections of vials to be digitized quickly because handling is minimal. It also reduces distortion of labels and specimens because placing the vials on their sides causes these objects to float down and rest against the glass. The main disadvantage of this approach may be the failure to expose/capture all label data if multiple labels are included in a vial and/or labels are oriented in such a way that the text cannot be seen. Also, in most cases, images of the specimens themselves will not be of high enough quality to facilitate species identification or morphological study. In some cases, single specimens from lots of larger invertebrate species (e.g., crustaceans) may be removed from jars and imaged next to jars and labels. More advanced 3-D imaging technologies may eventually provide the means to capture and segment undistorted images of fluid-preserved specimens and labels in situ, although vials containing numerous individual specimens and/or labels will continue to present difficulties.

### Invertnet mass digitization workflow

Combining the hardware and software technologies described above, InvertNet will implement a semi-automated workflow that is user-friendly, requires minimal training for the end user, and meets the goals of reducing the per-specimen cost of invertebrate collection digitization while minimizing risk of damage to the specimens. Design of the InvertNet workflow and user interfaces is underway and is addressing several important points.

**1. Ease of use.** Given the anticipated heavy use of the system by non-skilled workers (e.g., students) the capture hardware operation and data input workflows will be required to minimize errors, verify correct inputs, and support corrective measures.

**2. High performance capture and input.** The overall workflow should not be impeded by capture hardware, client-side processing, or data transfer. The operator should be able to work in a sustained manner.

**3. Fault resilience.** The workflow should not be impeded by transient network conditions between the worksite and the InvertNet website, which can manifest as network delay, connection failures, and off-line operations.

**4. Security.** Data, raw and processed, should not be lost in the capture and upload process and should be transferred from the capture site into secure storage as quickly as network connectivity permits.

**5. Flexibility.** The workflows and hardware should be adjustable to site-specific preferences such as batch processing, variations due to collection attributes, and in general be flexible.

**6. Maintainability.** The systems in participating sites will run identical software releases, be remotely supported and upgradable, and consistent across sites in hardware and software versions.

To support these goals we are implementing the following generic workflow:

**1. Capture Workstation Preparation.** Stage drawers to be digitized, power up capture station, preform calibration operation.

**2. Capture Operations.** Operator selects among capture recipes, inserts prepared drawer, initiates capture. When capture is complete, operator reviews real-time processed images for completeness and accuracy.

**3. InvertNet Login.** Operator logs into digitization software/portal within InvertNet “Digital Collections” space.

**4. InvertNet Input.** Operator creates capture record for each tray processed above, providing appropriate metadata into system, with automation support to avoid entering redundant data.

### Cyber-infrastructure

Providing access to large digital collections of invertebrate specimens will require a robust, Internet-based, information technology (IT) infrastructure to store and provide access to the data and images via the Internet, and ensure that access to the data is maintained over the long term. To do this, InvertNet has implemented a cloud based infrastructure based on the open source cloud project OpenStack (http://openstack.org). This allows the InvertNet website and databases to be mirrored across web servers at multiple locations, which yields faster response times for users of the website and allows for rapid and complete disaster recovery.

### Website and content management system

**Figure 2.**
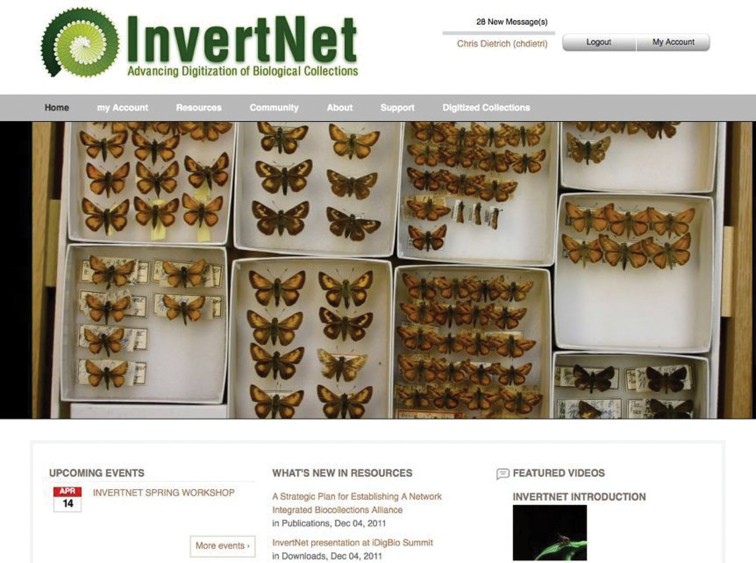
Current HUBzero-based InvertNet homepage showing top menu bar with content areas accessible to registered users.

The InvertNet web site (Fig. 2) is built on a robust cyberinfrastructure platform called HUBzero. HUBzero was developed with NSF support and designed specifically to support the kinds of large-scale, massively collaborative scientific research platforms that the ADBC program aims to build. HUBzero was originally designed to support a large community of nanotechnology researchers, but has since been adopted by a wide variety of other communities of researchers. The main advantage of HUBzero over other open-source content management systems is that it integrates a traditional CMS (Joomla; (http://www.joomla.org/) with powerful and highly customizable tools for data sharing, data analysis, data archiving. This gives InvertNet the ability to customize both back-end and front-end components of our cyberinfrastructure to meet our users’ needs for ingesting, processing, and visualizing digitized biological collections that include both traditional occurrence data and high-resolution graphics.

For example, to provide redundancy and preservation of contributed digital collections, we integrated HUBzero with an extensible cloud storage infrastructure (http://openstack.org), which allows us easily to scale up storage as the number of contributed collections increases, as well as spread storage resources over multiple redundant sites, improving security.

**Figure 3.**
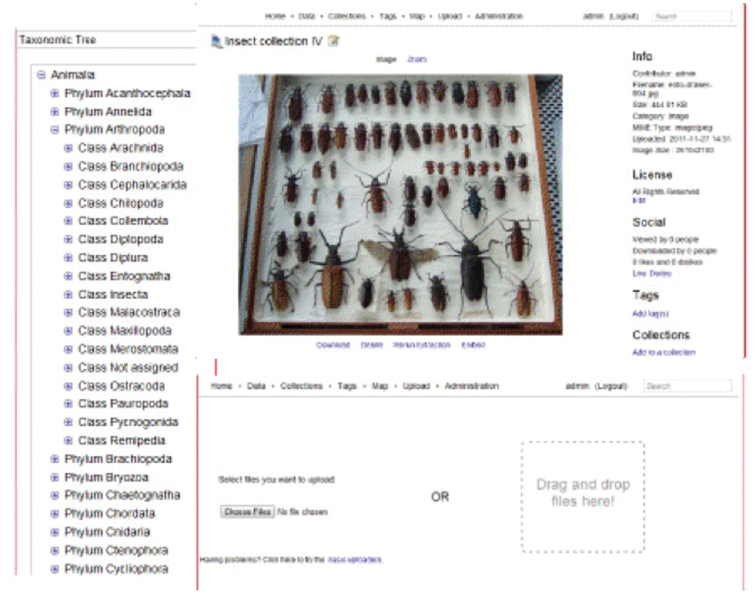
Current version of InvertNet’s Medici multimedia semantic content management system interface, accessible from InvertNet digital collections tab on homepage, showing taxonomic tree, drag and drop file upload space, and zoomable user interface for viewing gigapixel images.

To facilitate ingest and management of large collections of specimen images and data, we integrated HUBzero with the Medici multimedia content management system (http://medici.ncsa.illinois.edu/). Medici is a flexible, extensible semantic system designed to support any data format and multiple research domains and contains three major extension points: preprocessing, processing and previewing. When new data are added to the system, whether directly via the web application or desktop client, or through web services, preprocessing is automatically off-loaded to extraction services in charge of extracting appropriate data and metadata. The extraction services attempt to extract information and run preprocessing steps based on the type of data. For example, in the case of images, a preprocessing step creates previews of the image and automatically extracts metadata from the image and assigns a persistent, globally unique identification (GUID). Medici allows users to manage and aggregate collections comprising distributed sub-collections, track internal processing of resources and the creation of derived resources, provide GUIDs for resources suitable for citation and export metadata in globally understood standard formats. It also enables users to use desktop analysis tools via a remotely hosted web service in concert with the knowledge-space (i.e., digitized collections) without having to deal with download, installation, licensing, etc. Medici’s web interfaces (Fig. 3) are highly customizable, which enables us to create custom forms for capturing various kinds of metadata for different collection objects (e.g., whole drawers of pinned specimens). By making the clients and preprocessing steps independent and using Resource Description Framework (RDF) as a common domain-neutral data representation, the system can grow and adapt to different user communities and research domains, HUBzero also supports the development and integration of data processing (e.g., image analysis) and analytical tools that will allow users to manipulate and analyze data directly within the InvertNet platform.

Coupling the Medici content repository system with HUBzero will enable InvertNet to act as a collaborative social platform that can scale effectively and allow for submission of image collections. It will incorporate the digitization workflows, image post-processing, databases, environments for community building and collaboration, analytical tools, developer tools, and tools for education and outreach. To our knowledge, no other platform or website/application combines all of these capabilities and features to date.

## A few words on specimen-level label data capture

**Figure 4.**
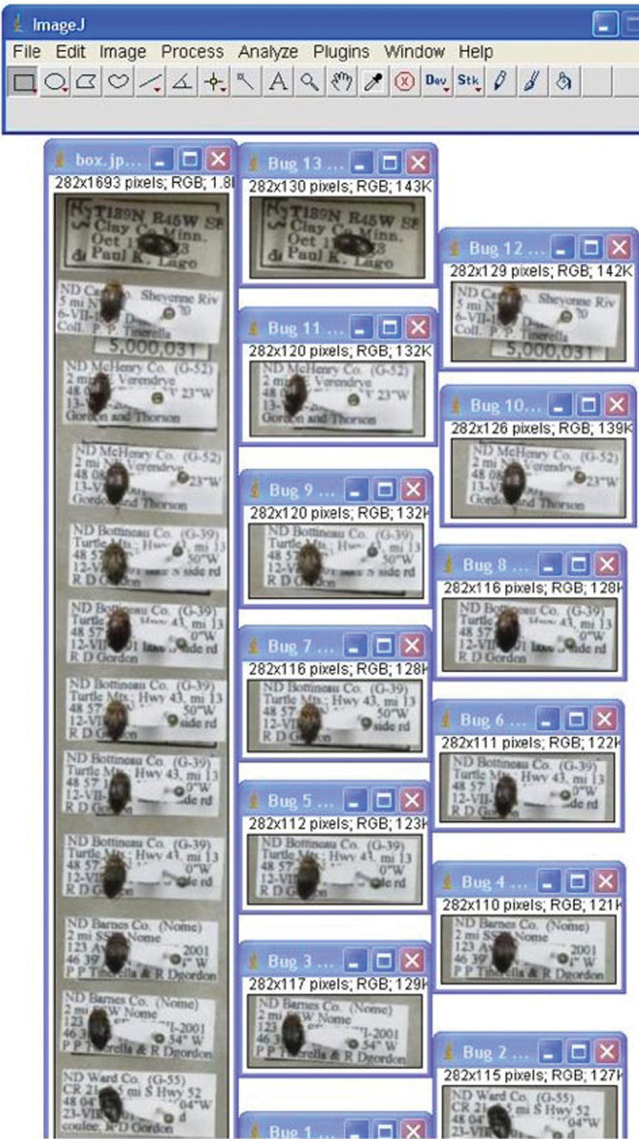
Image of multiple pinned insect specimens in unit tray (left) and same specimens segmented into separate files (right) using customized ImageJ image processing protocol.

As already demonstrated by the NCSU GigaPan project, use of advanced imaging techniques can provide rapid access to large numbers of invertebrate specimen images and the variety of potential uses of such images in research and education have only begun to be explored. Nevertheless, current biological collection database standards require capture of data at the specimen level. Images of entire storage units (e.g., drawers) may be segmented using image analysis software with the images of individual specimens placed in separate database records (Fig. 4). Because most insect specimens are small, labels pinned beneath them are often visible and, if the image quality is sufficient, the text of such labels may be read and interpreted. Thus, at least partial specimen occurrence and taxonomic data may be obtained directly from the images of many specimens. Even more advanced image capture and reconstruction techniques than those produced by the GigaPan or SatScan systems, including those being incorporated into the InvertNet digitization workflows, should provide even greater access to specimen-level label data, given the capability these techniques provide for viewing specimens from multiple perspectives. However, attempts to further automate the process of reading and interpreting specimen labels have, so far, had mixed success. The performance of available optical character recognition (OCR) software tested so far on insect specimen labels is generally poor. In most cases, more time must be spent detecting and correcting errors than would be required simply to enter the data into the appropriate fields of a database by hand. At present, the crowd-sourcing/citizen science approach to label data capture (Hill et al. 2012, this volume) appears to be the most promising avenue for entering such data as text into a relational, standards-compliant database. We anticipate that, by combining our advanced imaging protocols with a crowd-sourcing approach to label data capture, InvertNet will be able to deliver specimen-level occurrence and taxonomic data for a high percentage of the specimens present in the insect collections being digitized, all without the need for handling individual specimens. Ultimately, we envision InvertNet providing a digitization toolkit and research platform available to the entire natural history museum community.

## References

[B1] Ball J Gross J Gryzmala T Nishida G Oboyski P Gillespie R Roderick G Will K (2011) Calbug: a case study of digitization challenges for Entomology collections. Entomological Collections Network, Reno, Nov 2011. http://www.ecnweb.org/dev/files/talks_ecn_calbug_kwill2.ppt

[B2] Beeler T Bickel B Beardsley P Sumner P Gross M (2010) High-Quality Single-Shot Capture of Facial Geometry. Proceedings of ACM SIGGRAPH, ACM Transactions on Graphics 29(3): 40.1–40–9. http://graphics.ethz.ch/publications/papers/paperBee10.php

[B3] Bertone MA Deans AR (2010) Remote curation and outreach: examples from the NCSU insect museum GigaPan project. Proceedings of the Fine International Conference on Gigapixel Imaging for Science, November 11–13 2010. http://www4.ncsu.edu/~ardeans/BertoneDeansFAFS.pdf

[B4] Blagoderov V Kitching I Simonsen T Smith VS (2010) Report on trial of SatScan tray scanner system by SmartDrive Ltd. Nature Precedings. hdl:10101/npre.2010.4486.1 http://precedings.nature.com/documents/4486/version/1

[B5] Eades DC Otte D Cigliano MM Braun H (2012) Orthoptera Species File Online. http://orthoptera.speciesfile.org/HomePage.aspx

[B6] Harman KJ Fitton M Honey MR Martin G (2011) The Linnaean Society’s insect collection: increasing access through digitization. http://www.linnean.org/fileadmin/images/Collections/LS_Entomology_poster_FINAL_PRESS.pdf

[B7] Haüser CL Steiner A Holstein J Scoble MJ (Eds) (2005) Digital imaging of biological type specimens: a manual of best practice. European Network for Biodiversity Information, Stuttgart, 309 pp. http://www.gbif.org/orc/?doc_id=2429

[B8] Heidorn PB (2011) Biodiversity Informatics. Bulletin of the American Society for Information Science and Technology 37: 38-44. doi: 10.1002/bult.2011.1720370612

[B9] Hill A Guralnick R Smith A Sallans A Gillespie R Denslow M Gross J Murrell Z Conyers T Oboyski P Ball J Thomer A Prys-Jones R de la Torre J Kociolek P Fortson L (2012) The notes from nature tool for unlocking biodiversity records from museum records through citizen science. In: Blagoderov V Smith VS (Ed). No specimen left behind: mass digitization of natural history collections. ZooKeys 209: 219–233. doi: 10.3897/zookeys.209.3472 PMC3406478 22859890

[B10] Johnson NF (2007) Biodiversity informatics. Annual Review of Entomology 52: 421-438. doi: 10.1146/annurev.ento.52.110405.091259 16956323

[B11] Johnson NF (2009) Insect biodiversity informatics. In: Foottit RG Adler PH (Eds). Insect Biodiversity. Wiley-Blackwell, Oxford, UK: 433-443. doi: 10.1002/9781444308211.ch18

[B12] Kjar D Patel M Klopfer M Kweskin M Schultz T (2012) Smithsonian formicid type database. http://ripley.si.edu/ent/nmnhtypedb/public/namelisttemplates/longoutput-namelist.cfm?publicconsumption=1&typeid=663

[B13] Rojo MG Garcia GB Mateos CP Garcia JG Vicente MC (2006) Critical comparison of 31 commercially available digital slide systems in pathology. International Journal of Surgical Pathology 14: 285-305. doi: 10.1177/1066896906292274 17041192

[B14] Tinerella P (2010) Automation of natural history collections and bioinformatics: rapid optical data acquisition and automated computerization at INHS. National Science Foundation Award Abstract #1132188. http://www.nsf.gov/awardsearch/showAward.do?AwardNumber=1132188&WT.z_pims_id=5448

[B15] Vollmar A Macklin JA Ford L (2010) Natural history specimen digitization: challenges and concerns. Biodiversity Informatics 7: 93-112. https://journals.ku.edu/index.php/jbi/article/view/3992

